# Protocol for the diagnosis of keratoconus using convolutional neural networks

**DOI:** 10.1371/journal.pone.0264219

**Published:** 2022-02-18

**Authors:** Jan Schatteburg, Achim Langenbucher

**Affiliations:** Department of Experimental Ophthalmology, Saarland University, Homburg, Germany; Singapore Eye Research Institute, SINGAPORE

## Abstract

Keratoconus is the corneal disease with the highest reported incidence of 1:2000. The treatment’s level of success highly depends on how early it was started. Subsequently, a fast and highly capable diagnostic tool is crucial. While there are many computer-based systems that are capable of the analysis of medical image data, they only provide parameters. These have advanced quite far, though full diagnosis does not exist. Machine learning has provided the capabilities for the parameters, and numerous similar scientific fields have developed full image diagnosis based on neural networks. The Homburg Keratoconus Center has been gathering almost 2000 patient datasets, over 1000 of them over the course of their disease. Backed by this databank, this work aims to develop a convolutional neural network to tackle diagnosis of keratoconus as the major corneal disease.

## I. Introduction

**Fig 1:**
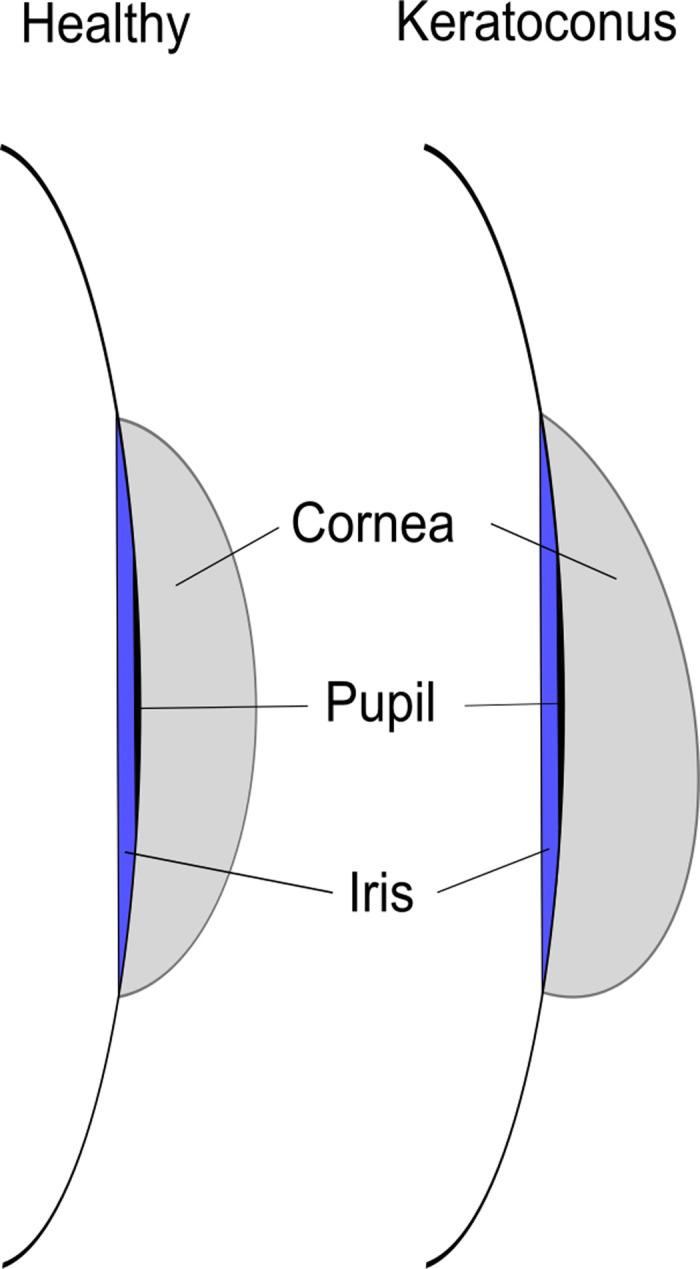
Schematic of a healthy eye and one with keratoconus.

Keratoconus is the most prevalent corneal disease with a reported incidence of 1:2000 [1], although it is acted on the assumption that the dark figures are considerably higher [2, 3]. It is a non-inflammatory ectasia that describes a conoid, asymmetrical deformation of the corneal center in combination with an eccentric thinning of the cornea (Fig 1). Keratoconus usually manifests bilaterally, although often one eye is more affected than the other. Symptoms in the early stage include diplopia (double vision) or Fleischer’s rings (iron dispositions). During further development, scars and obfuscation, tears in the Descemet membrane (epithelial basement), breaks in Bowman’s layer, and Vogt’s lines join the clinical picture. In an advanced stage a corneal edema can indicate an acute keratoconus [1, 4].

There are several ways to describe the three stages of keratoconus. The first classification was proposed by Amsler and was based on clinical features [5, 6]. Since then, multiple grading systems were developed. These parameters include the keratometry value (K value) describing the curvature of the cornea, the I-S value measuring the difference in keratometric power between the inferior and the superior hemispheres, the keratometric astigmatism index (AST) quantifying the degree of corneal astigmatism, corneal eccentricity, central corneal thickness, and many more. These often get combined into advanced parameters like KISA or KPI, which in turn are combined by diagnostic programs in order to mitigate the still present flaws of the former [7–10].

Therapeutic treatment of keratoconus includes the prescription of glasses or contact lenses in the early stages, implantation of corneal rings or segments of them, or corneal crosslinking at an advanced stage. In the final stage, only corneal grafting remains as a feasible treatment [1, 11].

### A. Diagnosis of keratoconus

For diagnosis, varying methods are applied. However, most advanced tools analyze backscattered light from the entire cornea to create 3D volume data. A common method for this is optical coherence tomography (OCT) [12].

Modern computer assisted diagnostic programs calculate parameters such as the curvature of the cornea or its thickness from the data, or a combined score of them, with some employing machine learning algorithms like the one by Maeda/Klyce [9]. However, they exclusively focus on the evaluation of parameters and, while analyzing most cases correctly, do not fully represent the clinical picture. This conclusion is supported by the fact that none of these parameters has been accepted as the undisputed gold standard [13, 14]. In order to solve these problems, an artificial neural network will be employed.

### B. Neural networks

Artificial neural networks are a type of machine learning models that are highly capable at solving a multitude of problems. Originally inspired by the biological neural networks that constitute animal brains, they consist of multiple layers of parameters (“artificial neurons”) [15]. The larger the number of layers is, the more advanced the extracted features from the input can be. In the example of image recognition, lower layers may identify edges, while higher layers may be able to distinguish a tear in a membrane from a scar. In general, each layer transforms the input data into a slightly more abstract and condensed version [16]. Common applications for artificial neural networks include computer vision (e.g. in autonomous driving), speech recognition, language translation, marketing, social networks, playing board and video games [17–21]. Only recently have advances been made into medicine [22].

### C. Convolutional neural networks

The origin of convolutional neural networks is found in the “neocognitron” introduced by Kunihiko Fukushima in 1982 [15]. They employ convolutional layers to extract features from data without the need to define which features to look out for. Therefore, their need for pre-processed data is low and they can solve tasks where not all key features have been discovered yet. Another advantage of convolutional neural networks is their unparalleled computation time for image processing that stems from the small number of connections per neuron in the convolutional and pooling layers. These special characteristics make convolutional networks the perfect fit for the analysis of 3D OCT images.

### D. Data preparation

One major point that can easily be overlooked during development is an appropriate selection of the training data. If a bias is present in the latter, the neural network will inherit it as well. This can lead to serious consequences [23, 24].

Once the data is selected, there are multiple ways to split up the data. This work will use k-fold cross-validation. Here, the data is split into k parts and k-1 parts are used for training while one part is left out for subsequential validation. After iterating over every permutation of training and validation sets, the network’s performance is examined on the test data to avoid a possible bias present in the training and validation data. The advantage of this technique is the reduction of overfitting. However, it requires additional computation time proportional to the number of repetitions (k) [25].

### E. Model training

During training the performance of a model is defined by a loss function that describes the error for one training example in order to minimize the error of the model during training. There are multiple possible loss functions to employ, depending on the posed problem. They commonly are margin-based, such as square loss, logistic loss or cross-entropy. The cost function then averages those over the entire training set [26].

After training has finished, the starting parameters of the network, also called hyperparameters, must be validated. These include the learning rate, the number of hidden layers, the number of neurons per layer and the convolution filter shape [27]. The learning rate refers to the step size towards the minimum of the loss function for each iteration of the network during training. The optimization algorithm most commonly used for this is gradient descent, where the step direction is the opposite of the gradient of the loss function. The step size can be constant; however, modern algorithms opt to change it between epochs. It decays over the learning process to avoid oscillation around a minimum and allow it to settle on it. Also, it gains momentum to speed up the learning process when the gradient of the loss function points in the same direction for a long time and also to make sure to overcome small local minima [28, 29]. The result is then backpropagated to the weights, which are modified along the gradient according to the chain rule. This requires the loss function to be differentiable [30–32].

When preparing the testing procedure, it is crucial to prevent overfitting. Overfitting describes adapting a model too close to the training data, to the point that it fails to properly assess input other than the one it was trained on. This means that natural variation is falsely considered a feature by the model, e.g. noise [33]. Besides a sufficiently large number of training data, common methods to reduce overfitting in neural networks are cross-validation, regularization, early stopping, dropout and model comparison [26, 34].

Even the best models would be worthless if artifacts mess with its prediction capabilities. Therefore, the network must be robust against small outliers without a significant reduction of its core function. Possible artifacts in an OCT graph include noise or possibly the signs of a different disease not incorporated in the trained model.

A model’s performance after training is determined with regards to four statistical evaluation metrics: accuracy (proportion of all correctly classified data), precision (proportion of predicted positives that actually is positive), recall/sensitivity (proportion of actual positives that is correctly classified as such) and specificity (proportion of actual negatives that is correctly classified as such) [35].

### F. State of the art of neural networks in diagnosis

As briefly mentioned in the introduction, neural networks have been employed in clinical ophthalmology for over 20 years now. However, clinical instruments have not exceeded the analysis of parameters. Only in 2020 successful research examining topographic images as a whole on keratoconus has been reported. Kuo et al. used three different types of convolutional neural networks to analyze color-coded topography maps recorded on a TMS-4 (Tomey Corporation). The data contained healthy eyes and ones with KC, plus a small portion of subclinical KC. They were augmented using rotation and shifting and were prelabeled based on central keratometry, I-S value, KISA%, and asymmetric bowtie presentation and produced high accuracies of 93% - 96% [36]. Zéboulon et al. used raw OCT topographic data recorded on an Orbscan to detect healthy eyes, ones with KC, and such with a history of refractive surgery. While they used 3000 data samples, examined multiple data types, and achieved over 99% accuracy, each data sample only contained 2500 pixels (equal to 50x50), which is rather low [37]. Elsawy et al. were able to distinguish dry eye, Fuchs’ endothelial dystrophy, and KC from healthy eyes using OCT images. They achieved over 99% accuracy with a pre-trained neural network [38].

However, none of these reports used full 3D tomographic OCT images, and while some have been able to detect subclinical KC, so far none have examined disease progression. The Homburg Keratoconus Center has been gathering data of over 1900 patients, many of them over the course of their disease, which provides an invaluable set of data for models aiming at detecting keratoconus in its early stage [39]. While the first stages of this work will focus on mere distinction of KC from healthy eyes, advanced versions are aimed to detect subclinical KC as well as distinguish it from other corneal diseases.

## II. Materials and methods

### A. Convolutional neural network

Neural networks have three different types of layers: input, output, and the layers between those, called the hidden layers. The neurons of subsequent layers are interconnected to varying degrees, transmitting their parameters via these connections to the next layer. The receiving neurons then offset these signals with their own parameters to transmit further on to the next layer [40, 41]. The functions used for these calculations are called activation functions. Radial basis function networks use the name giving functions for function approximation, with common ones being Gaussian functions, multiquadratic functions, or polyharmonic splines [42]. Max functions are used in the pooling layer of convolutional neural networks and pass the maximum of their input values. They are used to highlight features in data that has mostly low input values. Alternatively, average or minimum functions can be employed if the data calls for it. In the output layer, it is common to employ a softmax function for multiclass problems, which normalizes the outputs to a probability distribution [26], or a max function for dual-class problems.

In the convolutional layer, a filter is applied to the input map, usually with a size of 3x3. It feeds its data to a convolutional function which multiplies each weight in the filter with the respective pixel of the image. The results are summed up and transmitted to the neuron of the next layer of the network that lies in the center of the filter. Its activation function is a rectified linear activation function, which returns the positive part of its argument. Neurons with this are therefore called rectified linear units (ReLU) [43]. Their advantages are their efficient computation, scale invariance and a good gradient propagation [44]. Convolutional layers reduce the number of connections of each neuron compared to fully connected layers, drastically lowering computation time and improving feature extraction, as well as generalization from its training data. Their main application is image recognition, as they are highly capable in terms of feature detection, which is crucial in image recognition. The low number of connections per neuron can also deal with the exponential growth per pixel [26, 45, 46].

**Fig 2:**
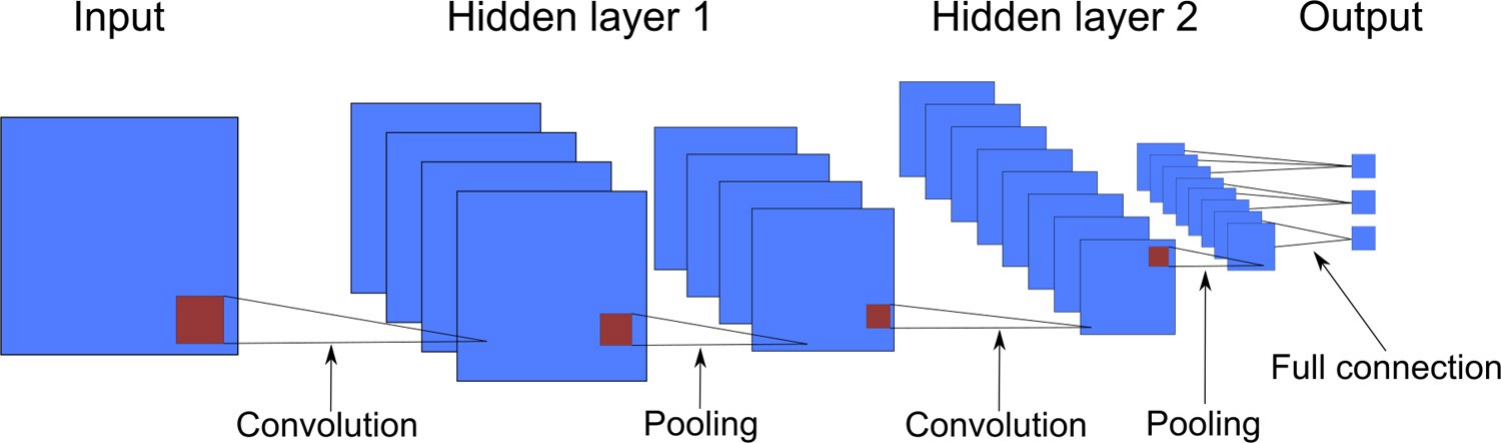
Schematic of a convolutional neural network.

Convolutional neural networks also use pooling layers, where the values of multiple neurons are condensed into one, usually in a 2x2 pattern. This is done to reduce computation time without compromising the quality of the results. The two common pooling types are max pooling and average pooling, transmitting the maximum and the average of each cluster of neurons to the next layer, respectively [47]. An example of a small convolutional neural network with two convolutional layers and two pooling layers can be seen below in Fig 2.

This study will employ three different network architectures. The first will consist of four blocks of a convolutional layer with 50 neurons and a size of the receptive field of 3x3, a max pooling layer, and a subsequent dropout layer (dropout of 20%) each. Those will be followed by two dense, fully connected layers of which the first one will employ ReLU as the activation function with 50 neurons. The activation function of the second dense layer depends on the problem posed: For the initial dual-class problem of distinguishing between keratoconus and no keratoconus, a max function will suffice. For more classes, a softmax function is needed to normalize the outputs to a probability distribution. The neuron count of the output layer will be equal to the number of classes present. The second architecture will consist of twelve blocks of convolutional and max pooling layers instead of four, with an equivalent neuron count. Both architectures will be compared to VGG16, which is a popular architecture for image classification [48]. For the analysis of 3D scans, a 3D convolutional function with a filter shape of 3x3x3 will be employed instead of the 2D function used for 2D data. While this will significantly increase computational time, it should not pose a problem since preliminary tests on 2D scans using an unrefined network point towards a computational time of 5–10 minutes per set of hyperparameters on a NVIDIA RTX 2060 Super graphics cards.

The optimizer function used is adam (adaptive moment estimation) as this has been widely accepted as common standard in image classification [49]. The split between training and validation data will be 80/20. The number of training epochs will initially be 10, however, this will be subject to change in order to prevent over- or underfitting. Overfitting can be identified if the validation loss is significantly higher than loss during training. Underfitting can be identified if validation loss still shows significant improvements by the end of the training epochs. Automated early stopping is an option that will be explored in this regard. Cross-validation will be performed 5 times. The great amount of data available will also add to the evaluation of the different models.

Investigations will take place on how the variation of the hyperparameters influences a network’s performance. For this, one or two layers of convolutional or dense, fully connected layers will be added after the initial tests have been performed. In addition to this, the neuron count per layer will also be varied to 10, 100 and 1000. The loss function will be mean squared error. Besides hyperparameters, performance of varying loss functions will be compared.

### B. Data gathering and processing

The data for the neural network will be gathered through two means: First, a pilot study with OCT data and automatic classification will be conducted. These datasets are already available in the Homburg Keratoconus Center anonymously gathered from over 1900 patients in the eye clinic of the Homburg University Hospital. The local ethics committee of the medical council of the Saarland (recognition number 157/21) has freed us from ethics approval for all the data. The data has been classified with parameters from the SS-1000 CASIA OCT Imaging System that recorded the data based on the ectasia screening index (ESI). An ESI of 30 is used as the breakpoint between eyes with keratoconus and ones without [50]. From these, preselection will take place so that an equal number of both will be available. Should this study prove to be promising, the same data but labelled as "healthy", "keratoconus", or "other diseases" by an ophthalmologist (fellow) who led the special consultations for ectatic corneal diseases for many years will be fed to the network and its performance on it will be studied, too. For later stages of this work, further diseases will be included in the data and their respective labels, too. The data from the Homburg Keratoconus Center consists of 3D OCT images of the entire anterior and posterior cornea, as well as the parameters calculated from this provided by the CASIA software. It is stored as csv files and dat files, respectively. The resolution of the CASIA is specified as 30μm transversely and 10μm axially [51]. The cornea maps each contain 32 * 400 data points, measured radially. For processing, the cornea maps will be normalized to a range of 0 to 1. From the pachymetric, topographic, and curvature maps the outer 30% of the data points will be cut off, leaving the relevant central area intact (97% of keratoconus cases are located in the central and paracentral region according to [52]. The exact cutoff percentage will be varied and results will be compared. The maps will not be centralized, turned, or tilted to ensure a robust feature detection. For first versions of the network, the data for training will be preselected by hand to exclude data with image errors like light reflections, for example. However, the ultimate goal is to apply it to all data, no matter its quality.

Initial implementations will focus on the examination of the thickness map. If the arbitrarily chosen accuracy threshold of 80% is reached, this will be repeated while including eye data with other diseases to ensure that the network is not forced to classify data as either "healthy" or "keratoconus". Subsequently, this procedure will be repeated on curvature and elevation maps, and their performances will be compared. If these three methods all show promising results, a program that combines the evaluations of all of them is planned. This could help dealing with inconclusive data by resolving those with the other maps.

Data augmentation will most likely not be needed, given the amount of available OCT scans. Should the need arise, however, the data will be augmented via shifting and rotation.

The neural network is being programmed in Python using the TensorFlow framework with the Keras library [53, 54]. These were developed specifically to be easy and fast to use, while providing full control and no loss of power versus programming everything by hand. For these reasons, it has been established as one of the top setups in the scientific community alongside Matlab.

As mentioned above, first test runs of an unrefined version analyzing 2D data point towards a computational time per set of hyperparameters of 5–10 minutes on a NVIDIA RTX 2060 Super graphics cards.

## III. Results

First, the convolutional neural network will be executed using the thickness maps that were classified automatically. The hyperparameters will be optimized, as well as activation and loss functions. Additionally, the cutoff percentage during pre-processing will be varied, and a run using unaltered data will be conducted. If the program performs promisingly (accuracy greater than 80%), this process will be repeated for curvature and elevation maps. Then the data will be augmented to include other diseases. A run with data augmented with subclinical keratoconus will be performed as well. Ultimately, a dataset containing healthy eyes, ones with fully manifested keratoconus, such with subclinical keratoconus, and eyes with other diseases will be fed to the neural network.

As an additional feature, heat maps of the areas that were most relevant in the assessment to the neural network will be reconstructed. The algorithm used to create these maps will be Grad-CAM [55].

## IV. Discussion

The performances on the varying types of data will be evaluated. Improvements will be made based on the results by adapting the hyperparameters, the activation functions, and the loss function. Analysis of the heat maps will be conducted by a medical expert. Any significant deviation of the impactful features from the assessment of the expert will be thoroughly inquired. They could signal a flaw in the program or indicate new features that experts have not yet put attention on. The impact of the data on the performance will be investigated, too, and adjustments to the pre-processing will be made, should they prove necessary. Of course, these will be restricted to equal treatment of all data to not include any bias. The neural network will finally be compared to the state of the art elaborated in the introduction.

## V. Conclusion

Convolutional neural networks are one of the most promising tools in diagnosis across all of medicine. A 3D analysis program for keratoconus would be a big help for doctors in corneal diagnostics. This work will aim to provide such a program. In the future, it can be augmented with an early detection system based on data of subclinical keratoconus, as well as the diagnosis of other corneal diseases.

## References

[1] Rabinowitz YS. Keratoconus. Survey of Ophthalmology. 1998; 42:297–319. doi: 10.1016/s0039-6257(97)00119-7 9493273

[2] Lang GK, Lang GE, editors. Augenheilkunde essentials. Stuttgart, New York: Georg Thieme Verlag; 2015.

[3] Godefrooij DA, Wit GA de, Uiterwaal CS, Imhof SM, Wisse RP. Age-specific Incidence and Prevalence of Keratoconus: A Nationwide Registration Study. American Journal of Ophthalmology. 2017; 175:169–72. doi: 10.1016/j.ajo.2016.12.015 28039037

[4] Krachmer JH, Feder RS, Belin MW. Keratoconus and related noninflammatory corneal thinning disorders. Survey of Ophthalmology. 1984; 28:293–322. doi: 10.1016/0039-6257(84)90094-8 6230745

[5] Amsler M. Le keratocone fruste au javal. Ophthalmologica. 1938; 96:77–83.

[6] Amsler M. Keratocone classique et keratocone fruste; arguments unitaires. Ophthalmologica. 1946; 111:96–101. doi: 10.1159/000300309 20275788

[7] Rabinowitz YS, McDonnel PJ. Computer-Assisted Corneal Topography in Keratoconus. Journal of Refractive Surgery. 1989; 5:400–8. 2488838

[8] Maeda N, Klyce SD, Smolek MK, Thompson HW. Automated keratoconus screening with corneal topography analysis. Invest Ophthalmol Vis Sci. 1994:2749–57. 8188468

[9] Maeda N, Klyce SD, Smolek MK. Neural Network Classification of Corneal Topography. Preliminary Demonstration. Invest Ophthalmol Vis Sci. 1995:1327–35. 7775110

[10] Rabinowitz YS, Rasheed K. KISA% index: a quantitative videokeratography algorithm embodying minimal topographic criteria for diagnosing keratoconus. Journal of Cataract & Refractive Surgery. 1999; 25:1327–35. doi: 10.1016/s0886-3350(99)00195-9 10511930

[11] Romero-Jiménez M, Santodomingo-Rubido J, Wolffsohn JS. Keratoconus: a review. Cont Lens Anterior Eye. 2010; 33:157–66; quiz 205. doi: 10.1016/j.clae.2010.04.006 . 20537579

[12] Fujimoto JG, Brezinski ME, Tearney GJ, Boppart SA, Bouma B, Hee MR, et al. Optical biopsy and imaging using optical coherence tomography. Nature medicine. 1995; 1:970–2. doi: 10.1038/nm0995-970 7585229

[13] Labiris G, Gatzioufas Z, Sideroudi H, Giarmoukakis A, Kozobolis V, Seitz B. Biomechanical diagnosis of keratoconus: evaluation of the keratoconus match index and the keratoconus match probability. Acta Ophthalmol. 2013; 91:e258–62. Epub 2013/04/05. doi: 10.1111/aos.12056 . 23557430

[14] Goebels S, Eppig T, Wagenpfeil S, Cayless A, Seitz B, Langenbucher A. Staging of keratoconus indices regarding tomography, topography, and biomechanical measurements. American Journal of Ophthalmology. 2015; 159:733–8. Epub 2015/01/26. doi: 10.1016/j.ajo.2015.01.014 . 25634534

[15] Fukushima K, Miyake S. Neocognitron: A Self-Organizing Neural Network Model for a Mechanism of Visual Pattern Recognition. In: Amari S, Arbib MA, editors. Competition and cooperation in neural nets. Proceedings of the U.S.-Japan Joint Seminar held at Kyoto, Japan, Febr. 15–19, 1982. Ed. by S[hōgo] Amari and M. A. Arbib. Berlin, Heidelberg usw.: Springer; 1982. pp. 267–85.

[16] Schmidhuber J. Deep learning in neural networks: an overview. Neural Netw. 2015; 61:85–117. Epub 2014/10/13. doi: 10.1016/j.neunet.2014.09.003 . 25462637

[17] Huval B, Wang T, Tandon S, Kiske J, Song W, Pazhayampallil J, et al. An Empirical Evaluation of Deep Learning on Highway Driving.; 07.04.2015. doi: 10.1039/c4lc01513d 25850799

[18] Deng L, Hinton G, Kingsbury B. New types of deep neural network learning for speech recognition and related applications: an overview. ICASSP 2013–2013 IEEE International Conference on Acoustics, Speech and Signal Processing. Vancouver, British Columbia, Canada, 26–31 May 2013; [proceedings. Piscataway, NJ: IEEE; 2013.

[19] Gentsch P. Künstliche Intelligenz für Sales, Marketing und Service: Mit AI und Bots zu einem Algorithmic Business–Konzepte, Technologien und Best Practices. Springer; 2017.

[20] Hazelwood K, Bird S, Brooks D, Chintala S, Diril U, Dzhulgakov D, et al. Applied Machine Learning at Facebook: A Datacenter Infrastructure Perspective. 24th IEEE International Symposium on High Performance Computer Architecture. Proceedings: 24–28 February 2018, Vienna, Austria. Piscataway, NJ: IEEE; 2018.

[21] Vinyals O, Babuschkin I, Czarnecki WM, Mathieu M, Dudzik A, Chung J, et al. Grandmaster level in StarCraft II using multi-agent reinforcement learning. Nature. 2019; 575:350–4. Epub 2019/10/30. doi: 10.1038/s41586-019-1724-z . 31666705

[22] Deo RC. Machine Learning in Medicine. Circulation. 2015; 132:1920–30. doi: 10.1161/CIRCULATIONAHA.115.001593 . 26572668 PMC5831252

[23] Tay Hunt E., Microsoft’s AI chatbot, gets a crash course in racism from Twitter. Guardian News & Media Ltd. 2016 [cited 13 Feb 2021]. Available from: https://www.theguardian.com/technology/2016/mar/24/tay-microsofts-ai-chatbot-gets-a-crash-course-in-racism-from-twitter.

[24] BBC. Google apologises for Photos app’s racist blunder. BBC 2015 [cited 13 Feb 2021]. Available from: https://www.bbc.com/news/technology-33347866.

[25] Kohavi R. A study of cross-validation and bootstrap for accuracy estimation and model selection. International Joint Conference on Artificial Intelligence. 1995; 14:1137–45.

[26] Goodfellow I, Bengio Y, Courville A. Deep learning. Cambridge, Massachusetts, London, England: MIT Press; 2016.

[27] Claesen M, Moor BD. Hyperparameter Search in Machine Learning.; 2015.

[28] Murphy KP. Machine learning. A probabilistic perspective. Cambridge, Massachusetts, London, England: The MIT Press; 2012.

[29] Ruder S. An overview of gradient descent optimization algorithms.; 2016.

[30] Rumelhart DE, Hinton GE, Williams RJ. Learning Internal Representations by Error Propagation. Cambridge, Massachusetts; 1985.

[31] Rumelhart DE, Hinton GE, Williams RJ. Learning representations by back-propagating errors. Nature. 1986; 323:533–6. doi: 10.1038/323533a0

[32] Werbos PJ. Backpropagation through time: what it does and how to do it. Proc IEEE. 1990; 78:1550–60. doi: 10.1109/5.58337

[33] Burnham KP, Anderson DR. Model selection and multimodel inference. A practical information-theoretic approach. 2nd ed. New York, NY: Springer; 2010.

[34] Yao Y, Rosasco L, Caponnetto A. On Early Stopping in Gradient Descent Learning. Constr Approx. 2007; 26:289–315. doi: 10.1007/s00365-006-0663-2

[35] Yerushalmy J. Statistical Problems in Assessing Methods of Medical Diagnosis, with Special Reference to X-Ray Techniques. Public Health Reports (1896–1970). 1947; 62:1432. doi: 10.2307/4586294 20340527

[36] Kuo B-I, Chang W-Y, Liao T-S, Liu F-Y, Liu H-Y, Chu H-S, et al. Keratoconus Screening Based on Deep Learning Approach of Corneal Topography. Transl Vis Sci Technol. 2020; 9:53. doi: 10.1167/tvst.9.2.53 . 33062398 PMC7533740

[37] Zéboulon P, Debellemanière G, Bouvet M, Gatinel D. Corneal Topography Raw Data Classification Using a Convolutional Neural Network. American Journal of Ophthalmology. 2020; 219:33–9. doi: 10.1016/j.ajo.2020.06.005 . 32533948

[38] Elsawy A, Abdel-Mottaleb M, Abou Shousha M. Diagnosis of corneal pathologies using deep learning. In: Manns F, editor. Opthalmic Technologies XXX. 1–2 February 2020, San Francisco, California, United States. Bellingham, Washington: SPIE; 2020. p. 80.

[39] Uniklinikum Saarland, editor. Augenklinik des UKS feiert 10 Jahre Homburger Keratokonuscenter (HKC). Uniklinikum Saarland 2020. Available from: https://www.uniklinikum-saarland.de/en/news/einzelansicht_news/aktuellesseite/article/universitaets-augenklinik-in-homburg-feiert-10-jahre-homburger-keratokonuscenter-hkc/.

[40] Winston PH. Artificial intelligence. 3rd ed. Reading, Mass.: Addison-Wesley; 1993.

[41] Jain AK, Mao J, Mohiuddin KM. Artificial neural networks: a tutorial. Computer. 1996; 29:31–44. doi: 10.1109/2.485891

[42] Orr MJL. Introduction to radial basis function networks.; 1996.

[43] Bengio Y. Learning deep architectures for AI. Boston: Now; 2009.

[44] Glorot X, Bordes A, Bengio Y. Deep sparse rectifier neural networks. Proceedings of the fourteenth international conference on artificial intelligence and statistics. pp. 315–23.

[45] Lawrence S, Giles CL, Tsoi AC, Back AD. Face recognition: a convolutional neural-network approach. IEEE Trans Neural Netw. 1997; 8:98–113. doi: 10.1109/72.554195 . 18255614

[46] Krizhevsky A, Sutskever I, Hinton GE. ImageNet classification with deep convolutional neural networks. Commun ACM. 2017; 60:84–90. doi: 10.1145/3065386

[47] Scherer D, Müller A, Behnke S. Evaluation of pooling operations in convolutional architectures for object recognition. International conference on artificial neural networks. 2010:92–101.

[48] Simonyan K, Zisserman A. Very Deep Convolutional Networks for Large-Scale Image Recognition.; 2014.

[49] Kingma DP, Ba J. Adam: A Method for Stochastic Optimization.; 2014.

[50] Tomey Corporation. CASIA2 Application Manual. Tomey Corporation [cited 26 Oct 2021]. Available from: https://simovision.com/assets/Uploads/User-Manual-Tomey-CASIA2-EN.pdf.

[51] Tomey Corporation. Fourier Domain OCT CASIA2. Tomey Corporation [cited 26 Feb 2021]. Available from: https://tomey.de/images/product_flyer/CASIA2_br_w.pdf.

[52] Wilson SE, Lin DT, Klyce SD. Corneal topography of keratoconus. Cornea. 1991; 10:2–8. 2019102

[53] Chollet F. Deep learning mit Python und Keras. Das Praxis-Handbuch vom Entwickler der Keras-Bibliothek. 1st ed. Frechen: MITP Verlags GmbH & Co. KG; 2018.

[54] Abadi M, Barham P, Chen J, Chen Z, Davis A, Dean J, et al. TensorFlow: A System for Large-Scale Machine Learning. 12th {USENIX} symposium on operating systems design and implementation ({OSDI} 16).

[55] Selvaraju RR, Cogswell M, Das A, Vedantam R, Parikh D, Batra D. Grad-CAM: Visual Explanations from Deep Networks via Gradient-Based Localization. Int J Comput Vis. 2020; 128:336–59. doi: 10.1007/s11263-019-01228-7

